# Accuracy of Individual Descriptors and Grading of Nodal Involvement by Axillary Ultrasound in Patients of Breast Cancer

**DOI:** 10.1155/2013/930596

**Published:** 2013-12-19

**Authors:** Navneet Kaur, Pradeep Sharma, Akhil Garg, Anupama Tandon

**Affiliations:** ^1^Department of Surgery, University College of Medical Sciences & GTB Hospital, University of Delhi, Delhi 110095, India; ^2^Department of Radiology, University College of Medical Sciences & GTB Hospital, University of Delhi, Delhi 110095, India

## Abstract

*Background*. Four-node sampling is a useful substitute for sentinel node biopsy in low resource settings. USG is being increasingly used as a preoperative tool to evaluate axilla. We conducted this study to assess the accuracy of different descriptors of axillary ultrasound and to formulate a model on grading of axillary involvement. *Material and Methods*. Thirty-four patients with clinically negative axilla underwent preoperative axillary ultrasound. The suspicious nodes were marked and details of various descriptors were noted. These nodes were sampled during axillary dissection and correlation of ultrasonographic findings with histopathological report was done to calculate accuracy of different descriptors. Based on this, a grading system of axillary lymph nodes involvement was formulated. *Results*. Based on the presence of various descriptors, five grades of nodal involvement could be defined. The most accurate descriptors to indicate nodal involvement were loss of hilar fat and hypoechoic internal echoes with specificity of 83% and positive predictive value of 92% each. The combination of descriptors of round shape with loss of hilar fat and hypoechoic internal echos had 100% specificity and positive predictive value. *Conclusions*. Grading of nodal involvement on axillary USG can be useful for selecting the most suspicious nodes for sampling during axillary dissection.

## 1. Introduction

Metastatic involvement of axillary lymph node is the single most important prognostic factor in breast cancer. The presence of axillary involvement in breast cancer determines the patient's survival and staging of the disease and plays an important part in local control. Until recently, axillary lymph node (ALND) dissection was considered as the reference method for detecting lymph node involvement. However the rate of axillary lymph node metastasis is very low in patients diagnosed at an early stage [1, 2]. Hence studies in the last decade discourage the use of ALND because of significant associated morbidities such as lymphedema, paresthesias, infection, and decreased range of movement of shoulder. To avoid these unnecessary morbidities the concept of sentinel node biopsy (SLNB) has gained increasing acceptance. Studies have proved that the drainage of breast lymphatics is in an orderly manner through initial node(s)—the sentinel lymph node (SLN) and almost 100% of breast cancer drain to axilla irrespective of the primary tumor's location [3]. Studies have proved that SLND alone is sufficient for management of axilla in early cancers if SLN is negative [4, 5]. However high setup and operating costs and complex techniques have limited the widespread application of this methodology.

Ultrasound has found an increasingly important place in the preoperative evaluation of clinically negative axilla. If USG detects axillary metastasis, the SLNB can be omitted and ALND can be performed directly. The sensitivity of USG in detecting metastatic involvement of axillary nodes has been reported to vary between 35% and 82%, while the specificity is between 73% and 98% [6, 7]. Some investigators reported that combined use of US and fine needle aspiration cytology (FNAC) could provide a highly accurate preoperative lymph node screening [8, 9]. However, FNAC is subject to sampling errors with reported false positive rate of 1.4% to 1.6% [10, 11] and also requires the cooperation of a highly experienced ultrasonologist and a cytopathologist.

The wide variation in reported sensitivity and specificity of axillary USG apparently results from the use of a number of descriptors defining nodal involvement by metastasis. However there has not been any attempt to assess the accuracy of individual descriptors so far. Hence we planned this study to evaluate the accuracy of individual descriptors (sensitivity, specificity, positive predictive value and negative predictive value, and diagnostic accuracy) when present alone as well as in combination.

As a second objective of the study, we tried to grade the nodal involvement based on sensitivity and specificity of various descriptors present either alone or in combination to enhance the accuracy of preoperative USG assessment of the axilla. This grading system can help the investigator to assess the axilla more accurately and also guide in choosing the most suspicious nodes for FNAC/core bx. USG guided localization of most suspicious nodes can also help the surgeons in sampling the axilla and provide a substitute for sentinel node biopsy in low resource settings.

## 2. Material and Methods

This prospective study was conducted on 34 consecutive patients of biopsy-proven breast cancer with clinically negative axillae in the Department of Surgery and Department of Radiology at UCMS & GTB Hospital, Delhi, India. All of these patients were planned to undergo definitive surgery, conservative or modified radical mastectomy with axillary clearance. Approval by the institutional ethics committee was obtained for the conduct of the study and patients were entered prospectively into the protocol of the study after signing an informed consent. The patients underwent a detailed clinical examination followed by examinations for baseline haematological and biochemical parameters including full blood count, blood urea, blood sugar, and complete liver function tests. They were subjected to other investigations for staging and metastatic workup as indicated, including mammography, chest X-ray, USG abdomen, bone scan, and CT scan.

All these patients were subjected to preoperative USG of the axilla to determine the presence of nodes which were sonographically suspicious of malignancy. The descriptors which were considered suspicious were A: size more than 10 mm; B: absence of fatty hilum; C: hypoechoic internal echo; D: circular shape; E: sharply demarcated border compared with surrounding fatty tissue; F: cortical thickening with eccentric lobulation of hypoechoic cortical rim [12–14]. The nodes which showed cortex more than 2-3 mm, but otherwise uniformly widened with a central echogenic hilum, were marked as benign nodes with reactive adenopathy.

**Figure 1:**
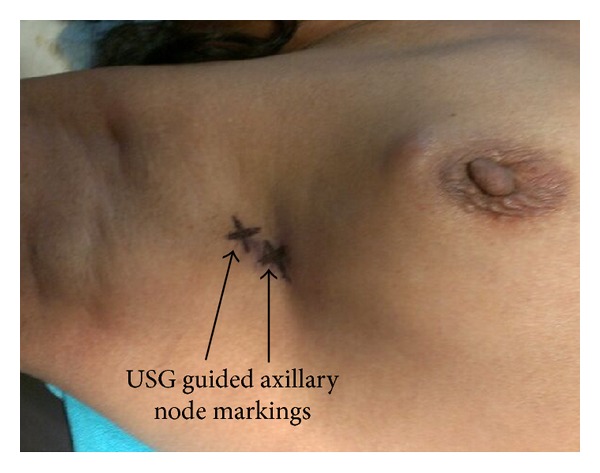
Marking of the suspicious nodes.

**Figure 2:**
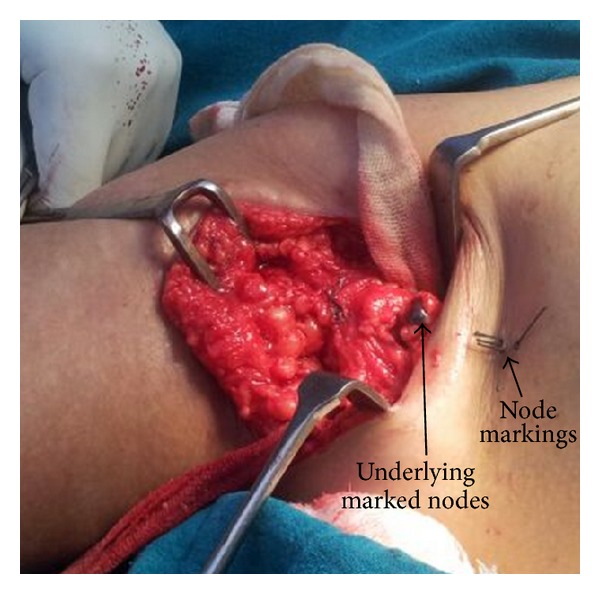
Sampling of the marked nodes.

Findings were noted with regard to the level and number of suspicious nodes. The details of descriptors characterizing findings of suspicious nodes in each case were also noted. These nodes were marked on the skin with an indelible marker and depth from the skin surface was also noted (Figure 1). At the time of surgery, after mobilisation of the axillary tail of breast, these nodes were taken out from the axillary fat pad separately, their localisation being guided by skin marking (Figure 2). In obese patients with abundant axillary fat it was difficult initially to locate the node, but with experience we overcame the problem. After the sampling of these suspicious nodes, axillary dissection was completed along with surgery for the primary lesion.

Lymph nodes dissected out of the axilla were subjected to histopathological examination for metastatic disease. Based on the correlation of USG and histopathologic (HPE) findings, sensitivity, specificity, positive predictive value (PPV), negative predictive value (NPV), and diagnostic accuracy were calculated for each descriptor alone and in combination. Based on these calculations, a grading system of ultrasonographic assessment of axillary lymph nodes was formulated.

## 3. Results

Thirty-four patients with biopsy-proven breast cancer with clinically negative axilla were included in the study. The youngest patient in this study was 27 years old and the oldest was 60 years old. Most patients (38.4%) were in the age group of 35–45 years. All patients were female. Upper outer quadrant was the most common location (70.6%) for primary lesion in the breast. As regards the tumor size, 8.8% had T1 tumor, 67.6% had T2 tumor, and 23.5% had T3 tumor. Around 87.5% of patients with lump size exceeding 5 cm were detected to have axillary metastasis on biopsy as compared to 33% in the group with lump size <2 cm.

Lymph nodes were demonstrated in 31 patients with preoperative ultrasonography of the axilla. In 3 patients no nodes could be identified. Since the cortices of normal nodes are isoechoic with the axillary fat, they may not be conspicuous with ultrasound unless the nodes can be identified by more echogenic hilar fat. In these 3 patients HPE was negative for axillary metastasis. In 13 patients nodes were radiologically benign with uniform widening of the cortex and echogenic hilum positioned symmetrically in the centre of the node. A total of 35 benign nodes were marked. None of these nodes had metastasis on HPE. Metastatic criteria were demonstrated in 62 lymph nodes in 18 patients. In 6 of these patients, despite the presence of criteria for metastatic lymph nodes on ultrasonography, histopathological examination did not reveal any lymph node metastases. Thus, ultrasonographic evaluation of the axilla in our study had a sensitivity of 100%, specificity of 72.7%, PPV of 66.7%, NPV of 100%, and a diagnostic accuracy of 85%.

A total of 97 nodes were marked in 31 patients. The size of nodes ranged from 0.4 cm to 2 cm (mean 0.97). Thirty-five nodes were marked with benign findings (size 0.4 to 1.6 mm, mean 0.75). Sixty-two nodes were marked for suspicion of metastasis (size 0.84 to 2 cm, mean 1.04). Of these, forty-four were positive for metastasis and 18 were negative. On further analysis of this data regarding the efficacy of individual predictors in predicting axillary nodal metastasis alone as well as in combinations, results are shown in Tables 1 and 2. Descriptor A had the highest sensitivity (81.8%) but lowest specificity (16.6%). Descriptor B and C almost always occurred together and thus had identical values and were most specific (83.3%) of all, with highest PPV (91.6%). Descriptor F had the lowest sensitivity and diagnostic accuracy and was also the least frequent finding on imaging.

When we looked at the combinations, the most accurate assessment was made when round lymph nodes were seen with loss of fatty hilum and hypoechoic internal architecture (Descriptors B + C + D) with specificity and positive predictive value of 100% each. Least specific combination was enlargement of the lymph node with asymmetric cortical thickening with specificity of 44.4% and PPV of 54.5%. The rest of the combinations had intermediate ranges of sensitivity and specificity as shown in Table 2.

## 4. Discussion

A wide range of sensitivity and specificity figures for axillary USG, using different descriptors, have been reported by various authors. For example Sousa et al. reported a 100% positive predictive value on finding of cortical thickness and nonhilar cortical vascular flow [15]. Verbanck et al. reported a sensitivity of 92% and specificity of 95% when using criteria of round or oval hypoechoic lesions with a diameter of 5 mm or more [16]. Oz et al. used the criteria of cortical thickening >3 mm, increased size of lymph node, an increase in sphericity index, increased cortical hypoechogenicity, and non hilar cortical flow and reported a sensitivity and specificity of 88.5% and 100%, respectively, and positive predictive value of 100% and negative predictive value of 66.6% [17]. Altinyollar et al. used criteria of loss of fatty hilum from central position, rounding of lymph node, decrease in echogenicity, and presence of eccentric cortical hypertrophy. They reported figures of specificity of 98.3%, sensitivity of 47.5%, positive predictive value of 95%, and negative predictive value of 73.7% [18]. Analysis of these reports shows that most commonly used descriptors of loss of fatty hilum, decrease in echogenecity and round lymph node have shown high sensitivity and specificity rates. Other less commonly used criteria have been cortical thickening, non hilar cortical vascular flow, increased size of lymph node, eccentric cortical hypertrophy, and sharply demarcated border.

In our study too, we found results on parallel lines. Our overall ultrasonographic evaluation of the axilla had a sensitivity of 100%, specificity of 72.7%, PPV of 66.7%, NPV of 100%, and a diagnostic accuracy of 85%. The high sensitivity rates in our study could be explained by the use of a wider range of descriptors compared to most of the other studies. This in turn brought down the specificity and PPV of the axillary ultrasound.

As regards our findings about the accuracy of individual criteria, Descriptor A (size more than 10 mm) was an unreliable predictor of malignancy and had high sensitivity (81.8%) but low specificity (16.6%) rates. The reported sizes of normal and metastatic lymph nodes are highly variable. Benign reactive enlargement of the lymph nodes can be seen in response to fibrocystic changes, infections, and any process such as a recent biopsy which may activate the reticuloendothelial system. In one large series, an average diameter of 6.5 mm was determined for uninvolved lymph nodes and 9.7 for metastatic nodes with a range from 1.8 to 40.6 mm [19]. Thus while larger lymph nodes tend to correlate with metastasis, even small lymph nodes, a few mm in diameter, may contain metastasis.

Descriptors B and C showed high specificity (83.3%) and PPV (91.61%) rates. They indicate the process of advanced nodal involvement and can be easily detected with USG. On invasion of the hilum, the lymph node acquires a homogeneous, hypoechoic pattern. Although the nodes' hilar fat may remain for some time, it gradually decreases over time. Most of the studies using these descriptors too have reported high rates of diagnostic accuracy [16, 18].

Descriptor D (round shape) also showed high specificity (83.3%) and PPV (87.5%) rates, though lower sensitivity, which apparently is due to the fact that change from normal oval to round shape happens only with advanced nodal involvement. Other studies too have reported high diagnostic accuracy of this descriptor [15–17].

Descriptors E and F showed lower rates of specificity and PPV and probably indicate intermediate patterns of metastatic involvement of the lymph node. Many metastatic nodes have smooth, sharp margins, as at times lymph node metastasis may grow quite large before they penetrate the capsule. Metastatic involvement may also displace the hilum to an asymmetric peripheral position with additional changes of asymmetric thickening of the cortical zone, seen on USG as hypoechoic or hyperechoic structural changes within the thickened, involved nodal cortex.

When we analyzed these descriptors occurring in combination, the descriptors of round shape with loss of hilar fat and hypoechoic internal echoes had 100% specificity and PPV. This combination can be considered almost diagnostic of metastasis. The next most predictive combination was enlargement of node with loss of fatty hilum and hypoechoic internal echos (specificity 88.8% and PPV 93.3%). These findings indicate a high suspicion of metastasis. Other less accurate combinations were enlargement of nodes with sharply demarcated borders or enlarged node with asymmetric cortical thickening and at best can suggest suspicion of metastatic involvement of the nodes.

Taking a cue from the BI-RADS-system for breast lesions based on analysis of combined features of different descriptors on breast ultrasound, different grades have been proposed, giving the highest level of suspicion for malignancy for a group of findings; the following grading system can be formulated for axillary nodal sonographic findings.

**Figure 3:**
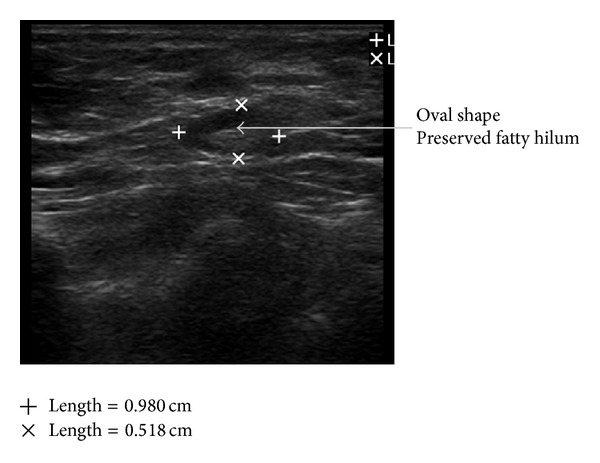
Benign node: oval shape, preserved central hilum.

**Figure 4:**
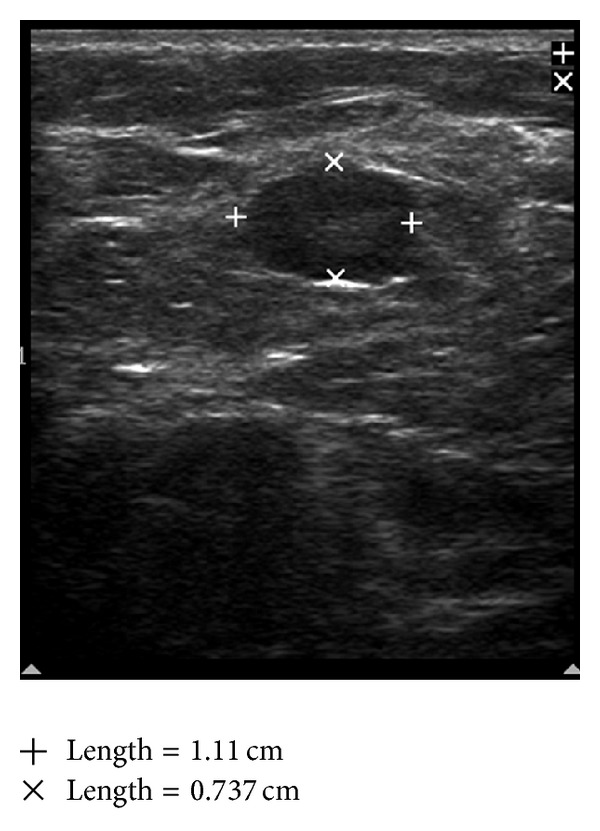
Suspicious node: sharply demarcated borders, asymmetric cortical thickening, and displaced hilum.

**Figure 5:**
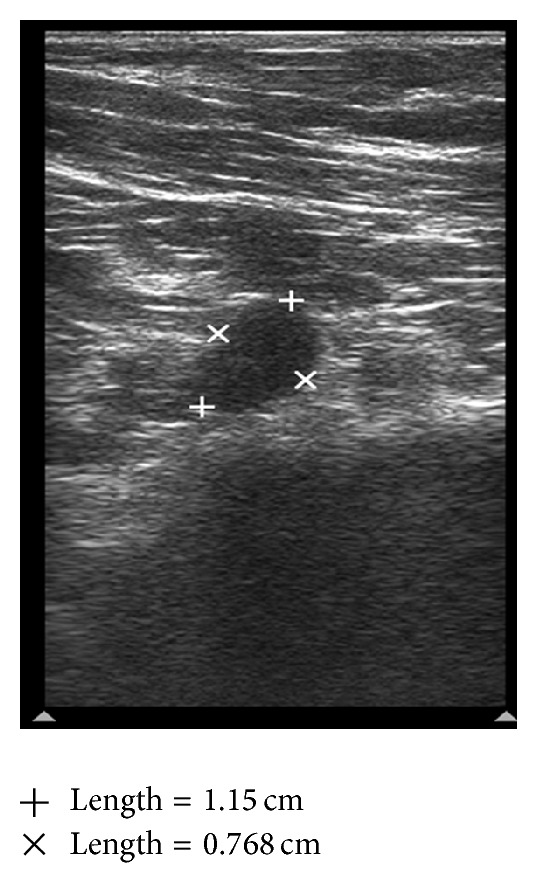
Suspicious node: cortical lobulation, cortical thickening, and displaced hilum.

**Figure 6:**
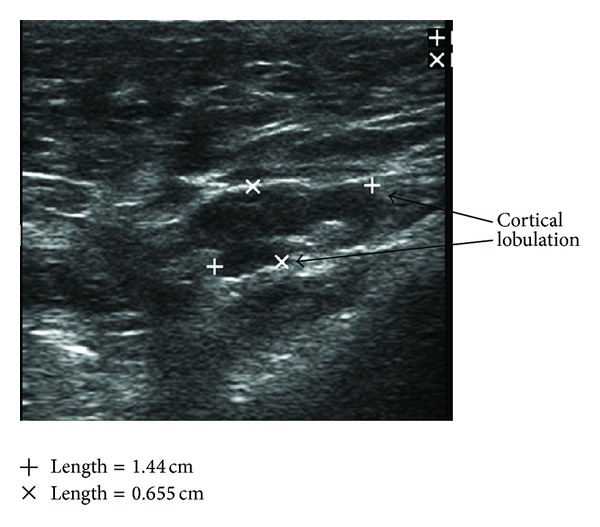
Highly suspicious node: enlarged node, cortical lobulation, and displacement of fatty hilum.

**Figure 7:**
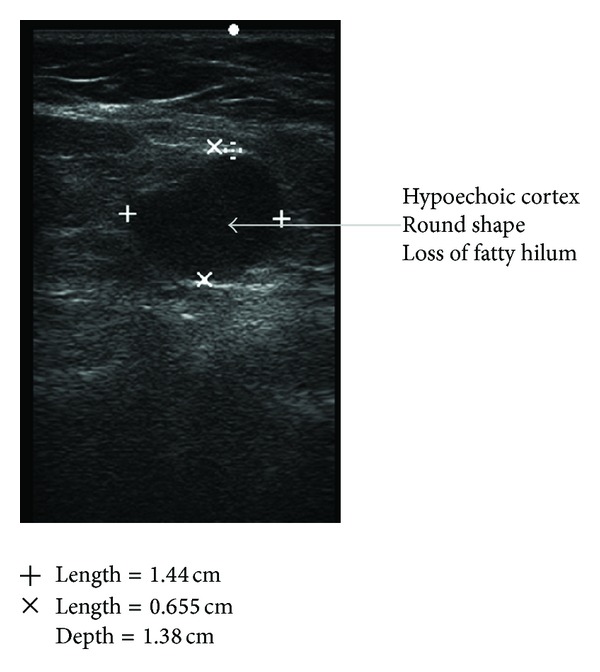
Involved lymph node: round shape, loss of fatty hilum, and hypoechoic internal echo.

 Grade I. Negative: no lymph node seen.
 Grade II. Benign findings: reactive lymph nodes with maintained anatomical feature of the node (Figure 3).
 Grade III. Suspicious: enlarged lymph nodes with asymmetric cortical thickening, cortical lobulations, sharply demarcated borders (Figures 4 and 5).
 Grade IV. Highly Suspicious for malignancy: asymmetric cortical thickening, hypoechoic internal echos, displacement of fatty hilum (Figure 6).
 Grade V. Involved: hypoechoic internal echos, loss of fatty hilum, round shape (Figure 7).

This grading system can be further refined by prospective application in a larger sample of patients and by evaluation of newer parameters such as non hilar cortical vascular flow as seen on Color Doppler, which has recently been reported to be a very sensitive descriptor [17]. Sonoelastography and further improvement in imaging quality may identify newer descriptors which may further enhance the accuracy of axillary USG.

This grading system can prove to be useful in selecting the appropriate lymph nodes for FNAC/core biopsy for preoperative assessment of the axilla. Nodes which show features of grade V or grade IV involvement should be targeted for sampling first over nodes of category III, if they are identified on sonogram. Four-node axillary sampling (4NAS) which is a cheaper alternative to sentinel node dissection relies on the surgeons ability to find abnormal palpable nodes in the axilla. To improve accuracy of 4NAS, blue dye has been found to be a useful complementary technique [20–22]. An ultrasound guided 4NAS where suspicious nodes can be identified for sampling based on their sonographic features may still prove to be a more cost-effective technique in the future.

## Figures and Tables

**Table 1:** Accuracy of individual descriptors to assess metastatic involvement of axillary lymph nodes.

Descriptor	Sensitivity	Specificity	Positive predictive value	Negative predictive value	Diagnostic accuracy
A	81.8%	16.6%	70.5%	20%	62.9%
B	70.45%	83.3%	91.61%	53.5%	74.69%
C	70.45%	83.3%	91.61%	53.5%	74.69%
D	63.6%	77.7%	87.5%	46.6%	67.74%
E	40.9%	77.7%	81.8%	35%	51.6%
F	50%	44.4%	54.5%	20%	32.2%

Descriptors: A: size more than 10 mm; B: loss of fatty hilum; C: Hypoechoic internal echo; D: round shape; E: sharply demarcated border; F: asymmetric cortical thickening with or without eccentric lobulation.

**Table 2:** Accuracy of combinations of predictors to evaluate the metastatic involvement of axillary lymph nodes.

Combinations of descriptors	Sensitivity	Specificity	Positive predictive value	Negative predictive value	Diagnostic accuracy
B + C + D	54%	100%	100%	47%	67%
A + B + C	63.6%	88.8%	93.3%	50%	70.96%
A + E + F	27.27%	77.7%	75%	30.43%	41.93%
A + E	40.9%	77.7%	81.8%	35%	51.6%
A + F	27.27%	44.4%	54.54%	20%	32.25%
